# Psychiatric Approaches for Disorders of Sex Development: Experience of a Multidisciplinary Team

**DOI:** 10.4274/Jcrpe.1044

**Published:** 2013-12-12

**Authors:** Burcu Özbaran, Samim Özen, Damla Gökşen, Özlem Korkmaz, Hüseyin Onay, Ferda Özkınay, Özgür Çoğulu, Serpil Erermiş, Sezen Köse, Ali Avanoğlu, İbrahim Ulman, Şükran Darcan

**Affiliations:** 1 Ege University Faculty of Medicine, Child and Adolescent Psychiatry, İzmir, Turkey; 2 Ege University Faculty of Medicine, Department of Pediatric Endocrinology, İzmir, Turkey; 3 Ege University Faculty of Medicine, Medical Genetics, İzmir, Turkey; 4 Ege University Faculty of Medicine, Department of Pediatrics, İzmir, Turkey

**Keywords:** Disorders of sex development, psychiatric disorders, gender identity

## Abstract

**Objective:** Disorders of sex development (DSD) are a group of congenital medical conditions that affect life as a whole. In this study, we aimed to reflect the experience of a multidisciplinary team in the clinical/psychiatric follow-up of a group of children and adolescents with DSD.

**Methods:** The study group consisted of 51 patients diagnosed with DSD. The Kiddie-Schedule for Affective Disorders and Schizophrenia, Wechsler Intelligence Scale for Children-Revised, Draw a Person Test and Children’s Apperception Test, and the Clinical Global Impression Scale (CGIS) were used for psychiatric evaluations.

**Results:** The mean age of the patients was 7.8 years (median: 7.8; min: 1.0; max: 18.0). Genetic evaluation showed 46,XX configuration in 15 patients (29.4%) and 46,XY in 35 (68.6%). One patient (2.0%) was diagnosed to have a sex chromosome disorder. Forty patients (78.4%) had no problems with their given gender identity and gender role. Thirty-four (66.7%) patients had normal intellectual capacity. Twenty-eight (54.9%) patients did not have any psychiatric problem. Depression, anxiety disorders, attention deficit/hyperactivity disorder, and adjustment disorders were the common diagnoses. The mean score of symptom severity on CGIS-severity-baseline was 6.15±0.68 and after one year, it was 1.46±0.51 (Z=-3.236 p=0.001). The mean score of CGI–Improvement was 1.23±0.44.

**Conclusion:** It is important to identify and treat the psychiatric disorders encountered in patients with DSD. A psychiatrist needs to be included in the professional team following these patients. Examination and observation results need to be shared by holding periodic team meetings to establish a wholesome point of view for every unique child.

**Conflict of interest:**None declared.

## INTRODUCTION

Disorders of sex development (DSD) are a group of congenital medical conditions which affect the whole life span (1). It is estimated that genital anomalies occur in one of every 4500-5000 births (2,3). The birth of a child with a DSD requires a long-term management strategy that involves a multidisciplinary professional team working with the patient and his/her family (4). The initial assessment of a patient with DSD requires a detailed maternal and family history as well as a careful physical examination (5).

Psychosexual development is traditionally conceptualized to have three components: gender identity, gender role, and sexual orientation. Gender identity refers to a person’s self assessment as male or female. Gender role describes the psychological and social characteristics that have sex-related variations within the general population, such as preferences in playing materials, clothing and behavior traits (such as physical aggression). Sexual orientation refers to the directions of erotic interest (heterosexual, bisexual, homosexual) and includes sexual behavior, fantasies, and attractions (4).

It is suggested that in humans, sexual behavior and sexual orientation are dependent on the intrauterine and postpartum organization of brain areas including the hypothalamus, the stria terminalis, the anterior commissural and mammillary nuclei, and the cortical areas. In patients with ambiguous genitalia at birth, the degree of masculinization of the genitals may not reflect the same degree of masculinization of the brain (6). Psychosexual development and sex orientation are also influenced by multiple factors such as exposure to androgens, sex chromosome genes, as well as by social variables and family dynamics (4).

Gender dissatisfaction denotes unhappiness with assigned sex. It is more frequent in individuals with DSD than in the general population. Causes of gender dissatisfaction are poorly understood, even among individuals without DSD. Karyotype, prenatal androgen exposure, degree of genital virilisation, or assigned gender are not reliable predictors (7,8,9,10).

In human behavior, it is possible to note some differences and variations in play behaviors and picture drawings of boys and girls. Women, flowers, butterflies in bright colors are more frequently encountered in drawings made by girls. Girls with congenital adrenal hyperplasia (CAH) were noted to show male drawing characteristics, even if the CAH was treated immediately after birth (11,12).

Studies on the psychiatric characteristics of children with DSD are scarce. Uslu et al (13) focused on type of rearing of children with male pseudo hermaphroditism and reported that children who were reared as boys were younger at time of referral, thus emphasizing the importance of early diagnosis.

In this study, we aimed to report the clinical follow-up results of children and adolescents who were referred to the multidisciplinary DSD team of the Ege University Faculty of Medicine Hospital, a team which includes pediatric endocrinology, pediatric surgery, genetics, and child and adolescent psychiatry clinicians. We focused on psychiatric diagnosis, psychiatric treatment when indicated, and gender satisfaction of the patients. 

## METHODS

Fifty-one children aged between 1 and 18 years (mean age: 7.83 years) who were referred with a DSD diagnosis to the consultation liaison psychiatry outpatient clinic of the Pediatric and Adolescent Psychiatry Department between 2004 and 2012 were included in the study.

One (2%) patient was diagnosed to have a 45,X/46,XY chromosome disorder; 10 (19.6%) patients had 46,XY disorders of testicular development; 25 (49%) were patients with 46,XY disorders of androgen synthesis or actions defect; 5 (9.8%) were patients with 46,XX disorders of ovarian development; and 10 (19.6%) were 46,XX fetal androgen excess patients. Following a complete description of the study and study procedure, the parents of all patients as well as patients over age 14 who were able to give informed consent were asked to provide written informed consent. Sociodemographic data were noted on a special data form prepared by the authors. All patients were assessed using the Kiddie-Schedule for Affective Disorders and Schizophrenia (K-SADS). K-SADS is a semistructured interview for determining the psychopathology of children and adolescents according to the DSM-IV criteria, and was developed by Kaufman et al (14). Gokler et al (15) adapted the method for use in the Turkish population. DSM-IV criteria were used for psychiatric diagnoses (16). Developmental and mental capabilities of children younger than 6 years of age were evaluated by using the Ankara Developmental Screening Inventory (ADSI). ADSI is a 154-item scale widely used in Turkey for the assessment and evaluation of social, motor, cognitive, and communicative levels of children aged between 0 and 6 years. The total development score reflects the general development level of the child, and it is obtained from the total of the four subscales (language-cognitive development, fine motor development, gross motor development, social interaction subscales). The Turkish version of the Wechsler Intelligence Scale for Children-Revised (WISC-R) was used for children older than 6 years. WISC-R is a standardized intelligence test for children and includes two parts - verbal and performance subtests. The Turkish standardization was made by Savasir and Erdogan-Bakar in 1995 (17,18,19).

Psychiatric symptom severity and improvement level after psychiatric follow-up and treatment were evaluated using the Clinical Global Impression Scale (Severity and Improvement) (CGIS/I) (20). CGIS was filled out in the first psychiatric evaluation of children by the child and adolescent psychiatrist. The scoring was: 1= not ill; 2= borderline ill; 3= slightly ill;

4= moderately ill; 5= markedly ill; 6= very much ill; 7= severely ill. CGIS was used for evaluating the treatment effect. CGIS-I was filled out 1 year after the first psychiatric evaluation.

The scoring was: 1= very much improved; 2= much improved; 3= slightly improved; 4= unchanged; 5= slightly worse;

6= much worse; 7= severely worse.

The classification of the medical diagnosis of patients with DSD was made according to the Lawson Wilkins Pediatric Endocrine Society and the European Society for Pediatric Endocrinology Consensus Statement (21).

The sociodemographic data form prepared by the authors was used to evaluate the economic status of the families, the academic performance of the patients, consanguineous marriages, as well as the medical diagnosis, raised gender, and karyotypes.

A psychiatric file including psychiatric examination and observation results, free play session and occupational therapy notes was prepared and recorded for each patient. All patients and families were evaluated by the consultation liaison team of the pediatric and adolescent psychiatry unit which comprises two psychiatrists, one psychologist, and one social worker.

During the interviews with the parents, the history of the disorder, the patient’s personality traits and patterns of behavior, gender roles, psychiatric symptoms, as well as the effect of the problem on the family members and family relationships were discussed. The interview with the patient aimed to assess the amount of information the child/adolescent had about his/her DSD diagnosis, to understand his/her feelings and thoughts as well as the social and academic difficulties he/she experienced. The psychiatric evaluation also included a description of gender identity, gender roles, and sexual orientation.

Psychiatric treatment included use of psychopharmacologic agents and supportive psychotherapy techniques as deemed necessary. All patients were followed at least in 10 individual sessions in the psychiatry clinic in addition to play room and occupational therapy sessions. Romantic relationships and sexual orientations were also noted in these sessions. Gender preference was determined after considering all observations including family interviews and psychiatric evaluations. The Goodenough-Harris Draw-a-Person Test was used for pictorial descriptions and perceptions of patients about themselves and others (13). The Children’s Apperception Test was also used as a projective test in psychiatric evaluations (22).

All parents and children were individually informed on the design of the study and signed an informed consent form in accordance with the Declaration of Helsinki.

**Statistical Analysis**

SPSS for Windows (version 16.0) was used for the statistical analysis.

## RESULTS

Twenty-seven (52.9%) patients were from low-, 23 (45.1%) were from middle-, and 2 (3.9%) - from high-income families. In 18 (35.3%) families, there was consanguinity between the parents. Nineteen (37.3%) children had one sibling, the median value of having a sibling was 1 (min: 0, max: 11). In the total series of 51 cases, one female and one male sibling (n=2) were found to have androgen resistance syndrome; two female siblings (n=2) had gonadal dysgenesis, and one female and one male sibling (n=2) had gonadal dysgenesis.

Twenty-four (47.1%) patients were in preschool, 13 (25.5%) in primary school, 6 (11.8%) in high school, and 8 (15.7%) patients had discontinued their school life.

The diagnoses of the patients according to the Lawson Wilkins Pediatric Endocrine Society and the European Society for Pediatric Endocrinology Consensus Statement are listed in Table 1. Four patients (7.8%) were diagnosed in the newborn period. The age at diagnosis of DSD was 4.83 years ± 6.25 (min: 0, max: 17.5).

According to the gender designation at birth, 26 (50.9%) patients were raised as a girl (8 patients 46,XX; 18 patients 46,XY), 25 (49.0%) as a boy (4 patients 46,XX; 20 patients 46,XY, 1 sex chromosome disorders). Eleven patients (21.56%) (2 patients 46,XX; 9 patients 46,XY) had uncertainty about their gender identity in the follow-up period; 4 of these had a psychiatric disorder diagnosis (Case 2, 8, 10, 13 in Table 2). Forty patients (78.4%) (11 patients 46,XX; 28 patients 46,XY; 1 patient with sex chromosome disorder) were stable on their gender identity and gender role, had a stable inner sense of being male or female, they did not have gender dysphoria; 9 of these patients had a psychiatric disorder. In 18 patients (35.29%) (5 patients 46,XX; 13 patients 46,XY), there was a discordance between karyotype and gender identity. However, of these 13 patients, those with 46,XY karyotype were happy with being a “girl” and patients with 46,XX karyotype were happy with being a “boy”. In thirty three patients (64.7%) (8 patients 46,XX; 24 patients 46,XY; 1 with sex chromosome disorder), karyotype and raised gender were in concordance (Table 3).

The conclusion of the DSD team for the patients was also recorded in their psychiatric files. Thirty-five patients (68.6%) (11 patients 46,XX; 23 patients 46,XY; 1 sex chromosome disorder) decided to continue in their raised gender that they were happy with. Ten patients (19.6%) (2 patients 46,XX; 8 patients 46,XY) also decided to continue their gender identity choice. Constructive genital surgery was planned for 6 patients (11.7%) (2 patients 46,XX; 4 patients 46,XY) (Table 3). A supportive psychotherapeutic treatment schedule was planned for all patients. Patients who were given a gender identity in the opposite sex, but who were dysphoric with that and wanted to make a change and demanded help for such a change, were supported by helping them change their lifestyle, including their way of dressing, their hair style, etc. Families were also supported. For patients who continued to their school life collaborative meetings with the school administrators were also arranged about changing their school uniforms, their adaptation to school environment, and their school performance. Cases 8 and 12 were our most difficult patients in that the adjustment period of their parents took a long time and because they were living in a small social circle; their social adjustment process was also difficult (Tables 2 and 3).

Levels of mental/intellectual capacity in these patients, their ages, and diagnosis are given in Table 4. Thirty-four (66.7%) patients had normal, 3 (5.9%) had borderline mental intellectual capacity, 11 patients (21.6%) had mild, and 3 patients (5.9%) had moderate mental retardation.

Twenty-eight (54.9%) of the patients did not show any psychiatric symptoms. Six patients (11.8%) had depression, 3 (5.9%) patients had anxiety disorder, 3 (5.9%) patients had attention deficit and hyperactivity disorder (ADHD), and one patient (2.0%) had adjustment disorder. One of the mild mentally retarded patients had also ADHD diagnosis and 3 patients had depression, besides their mental retardation. The mean score of CGIS-S baseline was 6.15±0.68 (min: 5; max: 7), while the mean score of CGIS-S after one year was 1.46±0.51 (min: 1; max: 2) (Z=-3.236 p=0.001); the mean score of CGIS-I was 1.23±0.44 (min: 1; max: 2) (Table 2).

Parents of all patients joined the parent psychoeducation sessions. Three (5.9%) of the patients were followed with supportive psychotherapy, without medication. Nine (17.6%) received antidepressant treatment, 1 (2.0%) patient received additional anxiolytic medication. One (2.0%) patient received additional antipsychotic medication because of his severe behavioral problems, and one patient (2.0%) was followed with psychostimulant medication (Table 2). 

## DISCUSSION

DSDs are rare disorders defined as discrepancy of chromosomal, gonadal, or anatomic sex (23). The optimal management of patients with DSD must be individualized and multidisciplinary (24). In this study, the psychiatric/clinical results on DSD patients, who are all still being followed by the multidisciplinary DSD team at a university hospital, were analyzed. We evaluated 51 patients followed for 8 years. Six (11.8%) patients were referred for surgery. Twenty-four (47.1%) of the patients were children of preschool age and 14 (27.4%) were adolescents. Early childhood and adolescence are complex developmental periods in human development, and clinicians working with these age groups should be aware of the developmental processes particular to these age groups. According to the separation individuation theory, children establish object consistency until age 3 and adolescence is the second individuation period in which the adolescents build their identity (1,25,26). It is important to prevent children and adolescents with DSD from getting a false reference and a false gender identity decision, which may also lead to an early decision for surgery. Surgery can relieve parental distress and improve attachment between the child with DSD and the family, but there are still controversies about the optimal timing of genital surgery. Some experts believe that decisions for appearance altering interventions should not be taken urgently and that it is more appropriate to delay surgery until a patient is old enough to be informed fully and provide consent for the intervention. However, there are also recommendations for genitoplasty to be performed between the 2nd and 6th months of life. Suggestions about the timing of surgery are controversial in the pertinent literature (24,27).

All patients presented in this report are still being followed by our DSD multidisciplinary team. In 10 (19.6%) patients, the team was not able to reach a final gender identity decision. Developing an inner sense of being a girl or a boy in childhood is a complex process; children go through psychological developmental periods until they can build a stable gender identity, as a part of identity formation (28). Psychiatric follow-up of children and adolescents with DSD includes a detailed psychiatric evaluation of the child and also of the family; it also includes identifying and treating comorbid psychiatric conditions as well as understanding the cultural and social environment of the child and his/her family (13).

Identifying a comorbid psychiatric disorder may help the clinician also to understand the brain development of the patient (29). A high proportion (54.9%) of our patients did not have any psychiatric diagnosis. It is important to treat the patients with any diagnosis of a psychiatric disorder for better treatment compliance in the endocrinology department and being without severe psychiatric complaints is also essential for building a stable gender identity. Six of the 13 patients in this series who had a psychiatric disorder were cases of CAH and were diagnosed to have depression and anxiety disorders. Previous studies on CAH patients also indicate that anxiety disorder and ADHD are frequently encountered in these cases (30). Studies on stress and quality of life level of CAH patients also suggest that these patients are under affective stress that may cause depression and anxiety disorders (31).

Twenty seven (52.9%) of the patients in this group were from low-income families. In the assessment and care of patients with DSD, along with the team of endocrinologists, the psychiatrist has a role in supporting the family and the patient, helping the child in his/her gender identity formation and also in identifying and treating psychiatric conditions such as anxiety, depressive disorders, ADHD which may accompany DSD. The social background of the patient also needs to be considered in the approach to the child and his/her family. There are suggestions in the literature that lack of nonprofessional psychological support may be a factor for negative influences on later sexual life (23).

In conclusion, the 8-year follow-up results of the DSD multidisciplinary team of a university hospital indicate that psychiatric consultation is important in the care of DSD patients. The findings also show that CAH patients are especially vulnerable to develop anxiety disorders and depression. It is important to follow these children and share the observations and clinical findings at periodic team meetings for establishing a wholesome point of view for every unique child.

## Figures and Tables

**Table 1:**
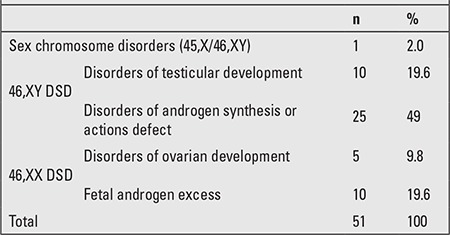
The diagnosis of the patients with disorders of sex development (DSD)

**Table 2:**
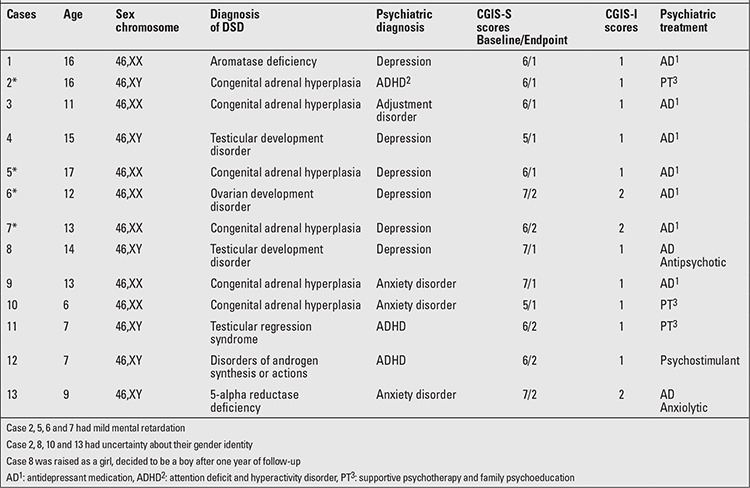
Age, medical and psychiatric features of patients with disorders of sex development (DSD) and their Clinical Global Impression Scale (CGIS) (Severity and Improvement) scores

**Table 3:**
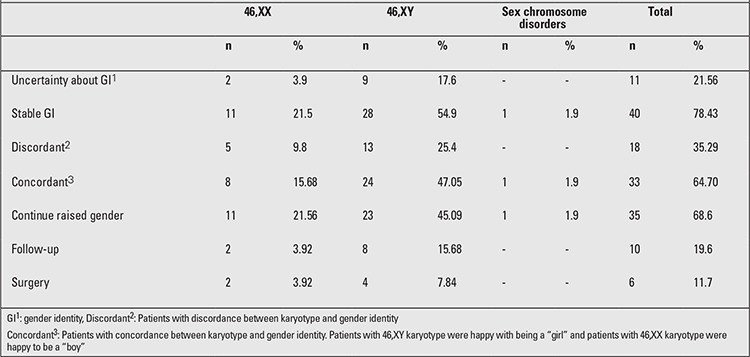
Follow-up results and decisions in gender guidance of disorders of sex development (DSD) patients

**Table 4:**
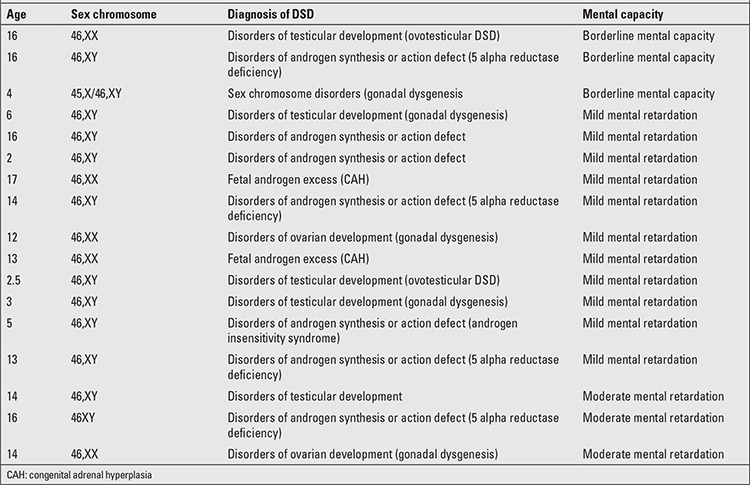
Mental capacity in this group of disorders of sex development (DSD) patients
